# Comparative Analysis of Nutritional Quality, Flavor-Active Amino Acids, and Chromatic Characteristics of Wild and Farmed Red Sea Bream (*Pagrus major*) Across Growth Stages

**DOI:** 10.3390/foods15132393

**Published:** 2026-07-05

**Authors:** Qisheng Zheng, Zhen Zhao, Qishuai Wang, Xinghong Luo, Ying Pan

**Affiliations:** 1 College of Animal Science and Technology, Guangxi University, Nanning 530004, China; 2 Key Laboratory of Aquatic Healthy Breeding and Nutrition Regulation of Guangxi Universities, Nanning 530004, China

**Keywords:** *Pagrus major*, cage-cultured, wild-caught, nutritional value, chromatic characteristics, amino acids, flavor, fatty acids

## Abstract

To address the quality divergence between wild and cage-farmed red sea bream (*Pagrus major*), we conducted a comparative analysis of their proximate composition, fatty acid and amino acid profiles, and chromatic characteristics across varying size and growth stages. Farmed fish exhibited substantial lipid deposition (peaking at 4.77% in F4), yielding a high relative percentage of n-3 long-chain polyunsaturated fatty acids. In contrast, wild fish maintained a leaner muscle profile (<2.43% lipid) and were uniquely characterized by an elevated abundance of C22:1n-9 (up to 11.52%), distinguishing them from farmed cohorts. Regarding sensory quality, large-sized wild specimens contained significantly higher concentrations of delicious amino acids (DAA, up to 8.83 g/100 g), particularly glycine and alanine, indicating a superior flavor profile. Colorimetric analysis demonstrated that wild fish maintained vivid reddish pigmentation in the caudal fin (a* = 8.73), whereas farmed fish exhibited marked skin darkening. These findings elucidate a distinct phenotypic and nutritional divergence: intensive farming enhances overall somatic lipid retention, while natural marine environments optimize flavor-active amino acid accumulation and visual appeal, providing critical baseline markers to guide precision aquaculture.

## 1. Introduction

Aquatic products serve as indispensable sources of high-quality protein and long-chain n-3 polyunsaturated fatty acids for humans, playing a pivotal role in ensuring global food security [1,2]. With the surging global demand for seafood and the continuous decline of wild fishery resources, aquaculture has emerged as the dominant industry to bridge the supply gap [3]. Consumer preference studies frequently indicate a perception that wild fish possess superior nutritional and sensory attributes compared to their farmed counterparts [4]. While empirical evidence demonstrates that farmed fish can be equally or more nutritious—particularly regarding n-3 polyunsaturated fatty acids—this persistent perception continues to directly influence the market value and consumer acceptance of aquaculture products [5]. Consequently, a systematic comparison of quality differences between wild and farmed fish has become a core research topic in the fields of aquatic and food sciences.

Fish muscle quality is a complex trait governed by the interaction between genetic background and environmental factors [6]. The distinct growth patterns of wild and cage-cultured environments fundamentally drive significant phenotypic differentiation. First, differences in exercise load directly determine the textural properties of the muscle. Wild fish engage in continuous swimming to evade predators and forage actively, whereas farmed fish have limited mobility in restricted environments. This disparity typically results in higher muscle fiber density and smaller fiber diameters in wild populations, leading to a firmer flesh texture [7]. Second, dietary composition is a decisive factor in the nutritional composition of the muscle. The nutritional composition of farmed fish largely mirrors their artificial formulated feed, with their fatty acid profiles directly reflecting the lipid sources in the diet [8,9]. In contrast, the nutrient accumulation in wild fish reflects the diversity of the natural food web. Furthermore, the physiological state of the fish, such as age, sexual maturity, and seasonal cycles, also dynamically regulates the allocation strategies of nutrients among muscle, liver, and gonads [10].

Red sea bream (*Pagrus major*) is a commercially important marine fish species in China, Japan, and South Korea. Due to its delicate texture, unique flavor, and rich nutritional profile, it is highly favored by consumers. For this economically valuable marine species in East Asia, body coloration is regarded as a critical criterion determining its commercial value and consumer preference [11,12,13]. Wild *P. major* typically displays a vivid reddish-pink hue due to the predation of astaxanthin-rich crustaceans. Conversely, cage-cultured individuals often exhibit dull or darkened coloration due to the lack of natural pigment sources and exposure to ultraviolet radiation in shallow waters. This phenomenon is highly similar to that observed in large yellow croaker, where a golden body color is a key quality indicator distinguishing wild from farmed populations [14]. Recent transcriptomic studies have further confirmed at the molecular mechanism level that wild fish typically exhibit more active gene expression in carotenoid metabolism and lipid transport pathways, which is directly correlated with pigment deposition efficiency and muscle textural properties [15]. Therefore, when evaluating the quality of *P. major*, investigating the variation in body coloration is as important as analyzing muscle nutritional composition.

Existing studies have confirmed these differences across multiple biochemical dimensions. In terms of proximate composition, the intake of high-energy commercial formulated diets and reduced physical activity in farmed fish typically drive elevated lipid deposition. This elevated lipid deposition is a defining characteristic distinguishing farmed fish from their leaner wild counterparts, often leading to significant differences in overall proximate composition [16]. Regarding fatty acid composition, farmed fish are often rich in linoleic acid (C18:2n-6) derived from terrestrial plant oils, whereas wild fish retain higher levels of arachidonic acid (C20:4n-6). Meanwhile, the content and ratios of EPA and DHA, which are crucial for human health, also show significant differentiation depending on food sources [17,18]. In terms of flavor, in addition to known differences in free amino acids and nucleotides [19], research based on odor fingerprints has pointed out that volatile flavor substances serve as important chemical markers distinguishing wild from farmed fish [20]. Current research on red sea bream has analyzed several key production traits and growth characteristics, yet systematic studies on nutritional components across different developmental stages remain largely unexplored. Therefore, elucidating the phased transformation of nutritional traits between farmed and wild red sea bream is an urgent task [21,22].

Although numerous studies have characterized the quality differences between wild and farmed populations of various aquatic species, such as *Solea solea* and *Scylla paramamosain* [23,24], knowledge regarding *P. major* remains predominantly limited to static, cross-sectional comparisons at harvest size. Current literature largely relies on single-timepoint evaluations and lacks a comprehensive cross-sectional perspective across the growth cycle. We hypothesized that wild and cage-cultured *P. major* would exhibit significant, stage-specific divergences in lipid deposition, fatty acid retention, and flavor-active amino acid accumulation throughout their developmental trajectories. To test this hypothesis, this study performed a systematic, multi-stage cross-sectional comparative analysis of wild and cage-cultured *P. major*. By evaluating proximate composition, fatty acid signatures, amino acid profiles, and chromatic phenotypes across different size classes, we aimed to delineate the specific biochemical differentiation driven by the rearing environment. Ultimately, these findings provide stage-specific phenotypic baselines to inform future feed formulation and aquaculture management.

## 2. Materials and Methods

### 2.1. Ethical Statement

All animal care and handling procedures in this study were approved by the Animal Ethics Committee of Guangxi University (Guangxi, China) and strictly adhered to the Guidelines for the Care and Use of Laboratory Animals.

### 2.2. Sample Collection and Preparation

The aquaculture experiment was conducted in the nearshore waters of Qiaogang Town, Beihai City, Guangxi, China (21°21′ N, 109°18′ E). The rearing system consisted of five net cages (6.5 m × 6.5 m × 5.0 m) deployed in a commercial farming area approximately 16 nautical miles offshore, with a water depth of 10–15 m. Each cage was equipped with a 40 mm mesh. Farmed *P. major* specimens (originating from hatchery-reared juveniles of captive broodstock) were obtained from these cages at five growth stages (3, 6, 9, 12, and 15 months; designated F1–F5, *n* = 100 per stage), with average body weights of 7.40 ± 2.01 g (F1), 40.84 ± 4.77 g (F2), 71.03 ± 14.08 g (F3), 234.82 ± 68.05 g (F4), and 300.05 ± 40.26 g (F5), respectively. While 100 individuals per stage were initially sampled to record basal morphometrics, all subsequent biochemical evaluations (including proximate composition, amino acid, and fatty acid profiling) were performed on six randomly selected independent biological replicates (*n* = 6) from each group to ensure analytical consistency.

**Figure 1:**
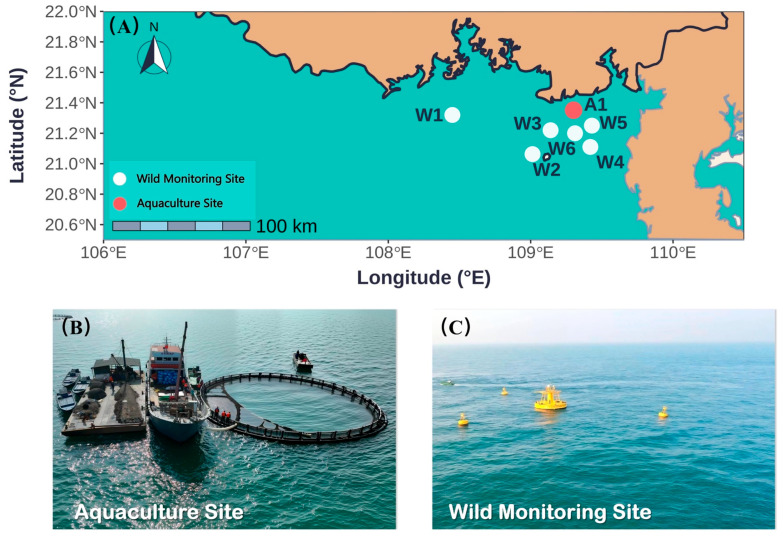
Spatial distribution of the sampling and aquaculture sites in the Beibu Gulf. (**A**) The map illustrates the geographical locations of the wild monitoring sites (W1–W6, blue circles) and the cage-culture aquaculture site (red circle) used in this study. (**B**) The inset photograph depicts the actual environment of the offshore net cages. (**C**) The inset photograph shows the open sea fishing grounds.

Conversely, acquiring precisely age-matched wild specimens is methodologically unfeasible due to the lack of absolute age indicators and the potential decoupling of size and age caused by environmental variations. To establish a comparable analytical framework, a size-class matching strategy was adopted. Therefore, while the term ‘growth stages’ may be used for comparative convenience in this study, for the wild cohorts it strictly denotes these size-matched classes rather than determined chronological age. Wild specimens (representing naturally recruited marine populations) were actively captured across six geographically proximate feeding grounds (W1–W6) within the same broad sea area (Figure 1). This multi-site sampling was strictly implemented to ensure the acquisition of a continuous spectrum of body weights corresponding to the developmental trajectory of the farmed cohorts, rather than tracing developmental migration.

A total of 103 wild individuals were categorized into six discrete size classes. The sample sizes and average body weights for the wild groups were W1 (*n* = 30; 4.90 ± 4.16 g), W2 (*n* = 41; 48.61 ± 14.12 g), W3 (*n* = 7; 67.51 ± 8.30 g), W4 (*n* = 13; 114.12 ± 16.35 g), W5 (*n* = 6; 247.74 ± 1.41 g), and W6 (*n* = 6; 517.37 ± 49.40 g). Upon collection, all specimens were euthanized via an overdose of MS-222 to minimize handling stress and subsequently transported to the laboratory on ice. The dorsal muscle was carefully dissected, skinned, and stored at −80 °C until further analysis. Consistent with the farmed cohorts, all subsequent biochemical evaluations for each of the six size classes (W1–W6) were performed on six randomly selected independent biological replicates (*n* = 6).

All sampling procedures for the cultured cohorts were conducted in the late part of March, June, September, December of 2024, and March of 2025. Wild specimens of various size classes were collected from the adjacent Beibu Gulf waters. Since *P. major* typically reaches sexual maturity at 2 to 3 years of age, the sampled individuals in this study were predominantly at sexually undifferentiated or immature stages. For the farmed group, fish were reared in offshore floating net cages (effective volume of approximately 2454 m^3^ per cage) with an initial stocking of 40,000 fish per cage. They were fed a commercial extruded pellet diet (Grobest Group, Fuzhou, China; containing ≥ 42.0% crude protein and ≥5.0% crude lipid) once daily at 14:00 to apparent satiation, achieving an average daily feeding rate of 3–4%. The basic hydrological parameters in the aquaculture area were continuously monitored throughout the cycle, with water temperatures ranging from 16.2 to 32.1 °C, salinity between 25 and 33, and dissolved oxygen levels maintained between 6.85 and 8.85 mg/L.

### 2.3. Chromaticity Measurement

**Figure 2:**
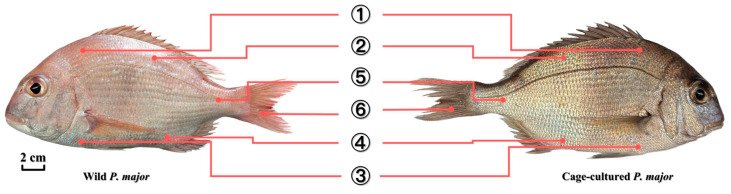
Schematic diagram of body surface color measurement sites for wild and cage-farmed cohorts of *P. major*. (Note: ① Back region A; ② Back region B; ③ Abdomen region A; ④ Abdomen region B; ⑤ Caudal peduncle; ⑥ Caudal fin).

In vivo chromaticity analysis of the body surface was performed immediately upon capture at the sampling sites. Prior to measurement, surface moisture on the fish was gently blotted dry using absorbent paper. Six specific anatomical loci were evaluated for each specimen: Back region A, Back region B, Abdomen region A, Abdomen region B, caudal peduncle, and caudal fin (Figure 2). Colorimetric parameters were quantified using a portable colorimeter (CR-10, Konica Minolta, Tokyo, Japan) based on the CIELAB three-dimensional color space. Prior to the measurements, the instrument was calibrated using the standard white calibration plate provided by the manufacturer. The recorded coordinates included L* (lightness, on a scale of 0–100), a* (redness-greenness coordinate; positive values indicate redness, negative indicate greenness), and b* (yellowness-blueness coordinate; positive values indicate yellowness, negative indicate blueness). To ensure accuracy and reproducibility, triplicate measurements were recorded at each designated locus, and the average values were calculated for subsequent statistical analysis.

### 2.4. Proximate Composition Analysis

The proximate composition of the muscle samples was determined strictly following the methods recommended by the Chinese National Food Safety Standards. Moisture content was measured gravimetrically by drying 2.0 g of muscle sample in an electric thermostatic drying oven (101-0AB, Taisite Instrument, Tianjin, China) at 103 °C to a constant weight, according to GB 5009.3-2016 [25]. Crude protein content was determined by the Kjeldahl method (GB 5009.5-2016) [26] using an automated Kjeldahl nitrogen analyzer (K1160, Hanon Advanced Technology Group, Jinan, China). Samples were digested with sulfuric acid, copper sulfate, and potassium sulfate at 420 °C prior to distillation and titration. Crude lipid was analyzed via the acid hydrolysis-solvent extraction method specified in GB 5009.6-2016 [27]. Briefly, samples were hydrolyzed with hydrochloric acid to release bound lipids, followed by extraction with diethyl ether and petroleum ether (boiling range 30–60 °C). Ash content was determined by incineration in a muffle furnace at 550 °C for 4 h, following GB 5009.4-2016 [28].

### 2.5. Amino Acid Profile Analysis

The amino acid composition was analyzed following the method recommended by the Chinese National Food Safety Standard (GB 5009.124-2016) [29] using two distinct hydrolysis procedures. For the determination of hydrolyzed amino acids (excluding tryptophan), samples were hydrolyzed with 6 M HCl and phenol in a sealed tube at 110 °C for 22 h under a nitrogen atmosphere. The hydrolysate was evaporated to dryness, redissolved in sodium citrate buffer (pH 2.2), and filtered through a membrane. The analysis was performed using an automated amino acid analyzer (LA8080, Hitachi High-Tech Science, Tokyo, Japan). For tryptophan determination, following the method recommended by the Chinese National Food Safety Standard (GB 5009.294-2023) [30], a separate alkaline hydrolysis step was conducted using 4 M LiOH at 110 °C for 20 h. The hydrolysate was neutralized to pH 6–8 with HCl, and tryptophan was quantified using a high-performance liquid chromatography system (Agilent 1200, Agilent Technologies, Santa Clara, CA, USA) equipped with a C18 column (4.6 × 150 mm, 5 µm) and a fluorescence detector (Ex 283 nm, Em 343 nm).

All amino acid quantifications were performed in triplicate. To evaluate potential flavor contributions, amino acids were categorized into four sensory groups: Umami Amino Acids (UAA: Asp + Glu), Sweet Amino Acids (SAA: Thr + Ala + Gly + Ser), Bitter Amino Acids (BAA: Val + Ile + Leu + Phe + His + Arg + Met), and Delicious Amino Acids (DAA: Asp + Glu + Gly + Ala + Arg).

### 2.6. Fatty Acid Profile Analysis

Fatty acid profiles were determined according to the acid hydrolysis-extraction method outlined in the Chinese National Food Safety Standard (GB 5009.168-2016) [31]. Briefly, samples were hydrolyzed in a water bath at 70–80 °C for 40 min using 8.3 mol/L HCl and 95% ethanol, followed by repeated extractions with a diethyl ether and petroleum ether mixture. For methylation, 20 mg of the extracted lipid was saponified with 0.5 mol/L sodium methoxide at 45 °C for 20 min, and subsequently methylated using a 14% boron trifluoride-methanol (BF_3_-MeOH) solution at 45 °C for an additional 20 min. The resulting fatty acid methyl esters (FAMEs) were then extracted into n-hexane.

Chromatographic analysis was performed using a gas chromatograph (GC-2014, Shimadzu, Kyoto, Japan) equipped with a flame ionization detector (FID) and a SIGMA SP-2560 capillary column (100 m × 0.25 mm × 0.2 μm). The injector and detector temperatures were maintained at 250 °C and 260 °C, respectively. Nitrogen was utilized as the carrier gas at a constant flow rate of 1.4 mL/min. The injection volume was 1 μL with a split ratio of 20:1 under a programmed temperature gradient. FAME peaks were identified by comparing their retention times against a 37-component FAME standard mixture (Supelco/Sigma-Aldrich, Bellefonte, PA, USA), and quantification was executed using the external standard method. Results were expressed as relative percentages of total fatty acids (%). All extraction and chromatographic procedures were performed in biological triplicates for each sample.

### 2.7. Nutritional Quality Evaluation

The essential amino acid composition of muscle protein was evaluated according to the FAO standard model for amino acids and Egg protein (FAO/WHO, 1991) [32]. The amino acid score (AAS), chemical score (*CS*), essential amino acid index (*EAAI*), and *F*-value were calculated as follows [33].

First, the content of each essential amino acid per gram of nitrogen (*AA*) was derived from the fresh sample data. As the initial measurements were expressed as a percentage of fresh weight, the values were converted using the following Equation (1):

(1) $$AA=\frac{aa}{CP}\times 6.25\times 1000$$

where *AA* represents the content of the specific essential amino acid in the sample (mg/g N); aa is the amino acid content in the fresh sample (%); CP is the crude protein content of the fresh sample (%); and the constant 6250 is the product of the nitrogen-to-protein conversion coefficient (6.25) and the unit conversion factor (1000 mg).

Based on the derived *AA* values, the Amino Acid Score (AAS) was calculated as a dimensionless ratio to assess the adequacy. of essential amino acids relative to the FAO/WHO reference pattern using Equation (2):

(2) $$\mathrm{AAS}(\%)=\frac{\mathrm{amino}\;\mathrm{acid}\;\mathrm{content}\;\mathrm{in}\;pagrus\;major\;(g/100\;g\mathrm{protein})}{\mathrm{amino}\;\mathrm{acid}\;\mathrm{content}\;\mathrm{in}\;\mathrm{FAO}\;\mathrm{reference}\;(g/100\;g\mathrm{protein})}$$

where FAO/WHO denotes the content of the corresponding amino acid in the FAO/WHO standard scoring pattern (mg/g N).

Simultaneously, the Chemical Score (CS) was determined as a dimensionless ratio to evaluate the protein quality relative to the whole Egg protein standard using Equation (3):

(3) $$CS(\%)=\frac{\mathrm{amino}\;\mathrm{acid}\;\mathrm{content}\;\mathrm{in}\;pagrus\;major\;(g/100\;g\;\mathrm{protein})}{\mathrm{amino}\;\mathrm{acid}\;\mathrm{content}\;\mathrm{in}\;\mathrm{egg}\;\mathrm{protein}\;(g/100\;g\;\mathrm{protein})}$$

where *AA*_(Egg)_ denotes the content of the corresponding essential amino acid in the whole Egg protein pattern (mg/g N).

To provide a composite assessment of protein quality, the Essential Amino Acid Index (*EAAI*) was calculated as the geometric mean of the ratios of all essential amino acids in the sample to their counterparts in the whole Egg protein using Equation (4):

(4) $$EAAI=\sqrt[{n}]{\frac{100t_{1}}{Q_{1}}\times \frac{100t_{2}}{Q_{2}}\times \cdot \cdot \cdot \frac{100t_{n}}{Q_{n}}}$$

where n is the number of essential amino acids considered; *t*_1_, *t*_2_ … *t_n_* are the contents of individual essential amino acids in the sample (mg/g N); and *Q*_1_, *Q*_2_ … *Q_n_* are the contents of the corresponding essential amino acids in the whole Egg protein pattern (mg/g N).

Finally, the F-value was calculated using Equation (5) to determine the ratio of branched-chain amino acids (BCAAs) to aromatic amino acids (AAAs):

(5) $$F=\frac{Val+Leu+Ile}{Phe+Tyr}$$

where Val, Leu, Ile represent the contents of Valine, Leucine, and Isoleucine (branched-chain amino acids) and Phe and Tyr represent the contents of Phenylalanine and Tyrosine (aromatic amino acids).

### 2.8. Statistical Analysis

All data are presented as mean ± standard deviation (SD). Statistical analyses were performed using SPSS 29.0 (SPSS Inc., Chicago, IL, USA), and data visualizations were generated using R Studio (version 4.3.1). Differences among experimental groups were evaluated using one-way analysis of variance (ANOVA) due to the non-orthogonal nature of the environmental variables between the wild and cultured groups. When significant main effects were observed, Duncan’s multiple range test was applied for post hoc comparisons. A significance level of *p* < 0.05 was considered statistically significant.

## 3. Results

### 3.1. Proximate Composition

**Figure 3:**
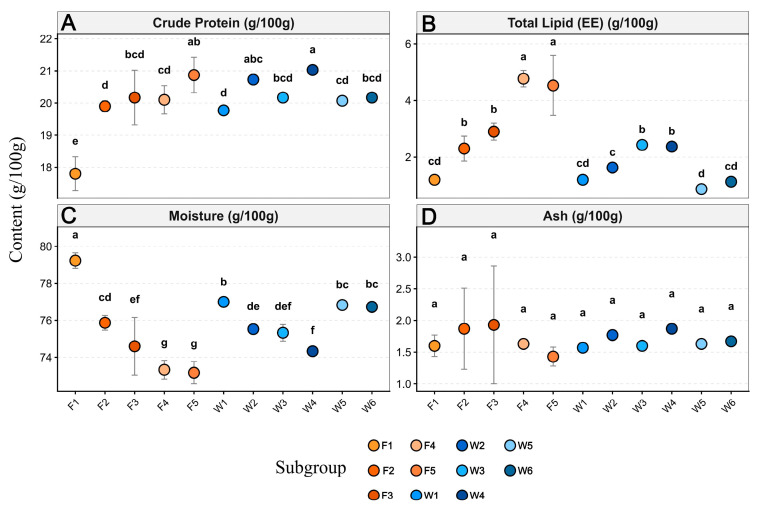
Comparative analysis of proximate composition between farmed and wild *P. major* populations throughout the growth cycle. (**A**) Crude protein content. (**B**) Total lipid content. (**C**) Moisture content. (**D**) Ash content. Data are presented as mean ± SD. Different lowercase letters above the bars indicate significant differences among groups (*p* < 0.05). The plots highlight the distinct lipid deposition strategy in farmed fish versus the lean profile of wild fish.

The proximate composition of *P. major* muscle, including moisture, crude protein, crude lipid, and ash, is summarized in Table 1 and visually depicted in Figure 3. Total ash content remained relatively stable across all groups, ranging from 1.43% to 1.93%, with no significant differences observed regardless of age or culture environment (*p* > 0.05). In contrast, crude protein content exhibited distinct variations. In farmed fish, muscle protein displayed a significant increasing trend with age, rising from a minimum of 17.80% at F1 to plateau at approximately 20.10–20.87% in the later stages (F3–F5). Conversely, wild fish maintained a consistently high protein level (>19.7%) throughout their life history. Notably, at the juvenile stage, the protein content of wild fish (W1, 19.77%) was significantly higher than that of the size-matched farmed group (F1) (*p* < 0.05). The highest protein content in the wild population was recorded in the W4 group (21.03%), while the values in the W3 and W6 groups were identical (20.17%).

Lipid and moisture contents displayed a pronounced inverse relationship, which was strongly influenced by the culture environment. Farmed fish were characterized by intense lipid deposition as the culture duration prolonged. The crude lipid content elevated dramatically from 1.20% in the F1 group to a peak of 4.77% in the F4 group. Statistical analysis indicated that the lipid levels in adult farmed fish (F4 and F5) were significantly higher than those in all wild groups (*p* < 0.05). Wild fish, in comparison, exhibited a much leaner profile, with lipid contents generally fluctuating between 0.87% and 2.43%. Mirroring the lipid trend, the moisture content in farmed fish declined significantly with age, reaching its lowest values in the F4 and F5 groups (73.17–73.33%). In contrast, wild fish maintained significantly higher moisture levels (>74.3%) across all sizes, reflecting the distinct metabolic and energetic strategies between the two populations.

### 3.2. Amino Acid Composition and Nutritional Evaluation

**Figure 4:**
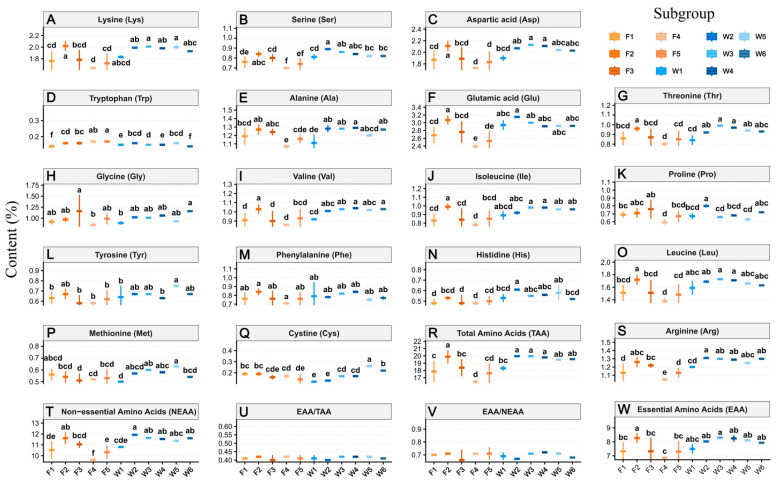
Dynamics of amino acid composition and nutritional evaluation indices. (**A**–**Q**) Profiles of individual amino acids (g/100 g fresh weight). (**R**–**W**) Comparative analysis of nutritional groups: (**R**) Total Amino Acids (TAA); (**S**) Arginine (Arg); (**T**) Non-essential Amino Acids (NEAA); (**U**) EAA/TAA ratio; (**V**) EAA/NEAA ratio; (**W**) Essential Amino Acids (EAA). Orange bars represent farmed groups (F1–F5), and blue bars represent wild groups (W1–W6). Error bars indicate SD. Different letters indicate significant differences (*p* < 0.05).

A comprehensive profile of 18 amino acids was identified in the muscle of *P. major*, comprising 8 essential amino acids (EAAs), 2 semi-essential amino acids, and 8 non-essential amino acids (NEAAs) (Table 2); the overall distribution and accumulation trends are illustrated in Figure 4. Glutamic acid (Glu), aspartic acid (Asp), lysine (Lys), and leucine (Leu) were the predominant amino acids across all groups; collectively, they accounted for over 40% of the total amino acid (TAA) content.

The amino acid accumulation patterns exhibited distinct ontogenetic variations between the two populations. In the farmed group, TAA and EAA contents followed a non-linear trend, peaking significantly at F2 with values of 19.88 g/100 g and 8.27 g/100 g, respectively. However, a marked decline was observed at F4, where TAA (16.46 g/100 g) and EAA (6.86 g/100 g) dropped to their lowest recorded levels. Although a slight recovery occurred at F5, the levels remained significantly lower than those at the 6-month peak (*p* < 0.05). In contrast, the wild population maintained consistently high amino acid concentrations after the juvenile stage. The TAA content in wild fish increased from 18.31 g/100 g in W1 to a plateau of approximately 19.5–20.0 g/100 g in the W2-W6 groups. Notably, the TAA and EAA contents in large-sized wild individuals (W5 and W6) were significantly higher than those in the size-matched farmed group (F5) (*p <* 0.05). Despite these quantitative variations, the ratios of EAA/TAA (0.40–0.42) and EAA/NEAA (0.62–0.72) in all groups satisfied the FAO/WHO ideal protein standards, confirming *P. major* as a high-quality protein source regardless of origin.

The protein quality was comprehensively evaluated using the Essential Amino Acid Index (*EAAI*) and F-value, as presented in Table 3, along with the Amino Acid Score (AAS) and Chemical Score (CS) (Table 4 and Table 5). Generally, an AAS or CS value close to or greater than 1.0 indicates that the essential amino acid requirement is fully met, whereas *EAAI* values above 75 denote high-quality protein. Lysine scores (AAS) consistently exceeded 1.5 across most groups, highlighting the species as an excellent source of lysine. However, tryptophan (Trp) was identified as the first limiting amino acid, with CS values ranging from 0.44 to 0.52. The *EAAI* values revealed a divergence in nutritional quality with age. Farmed fish exhibited high *EAAI* values (>85) during the early stages (3–6 months) but experienced a significant reduction with prolonged culture, dropping to 72.75–73.75 in the adult stages (F4–F5). Conversely, wild fish demonstrated superior nutritional stability in later growth stages, with the highest *EAAI* recorded in the W5 group (85.14), which was significantly higher than that of the synchronous farmed fish (F5, 73.75) (*p <* 0.05).

Regarding the *F*-value (the ratio of branched-chain amino acids to aromatic amino acids), the calculated values for all experimental groups ranged from 2.34 to 2.54. Specifically, the wild population maintained F-values between 2.42 and 2.54 in the adult stages (W4–W6). In comparison, the farmed group exhibited slightly lower values ranging from 2.34 to 2.37 during the later growth stages (F4–F5).

### 3.3. Flavor-Related Amino Acid Composition

The composition of flavor-related amino acids (UAA, SAA, BAA, and DAA) is summarized in Table 6.

The content of Umami Amino Acids (UAAs, including Asp and Glu), which contributes to the characteristic savory taste, exhibited significant differences. The wild group maintained a stable high level of UAAs (4.84–5.22 g/100 g), generally correlating with the high protein content observed. Notably, Glutamic acid (Glu), the most critical umami component, was consistently abundant. In the adult stage, the UAA levels in wild fish (e.g., W6: 4.94 g/100 g) were significantly higher than those in the synchronous farmed group (F5: 4.37 g/100 g) (*p* < 0.05).

The content of Sweet Amino Acids (SAA, including Thr, Ala, Gly, and Ser) accounted for a substantial proportion of the total amino acids. Wild fish generally possessed higher SAA levels. Specifically, Glycine (Gly) and Alanine (Ala) were the dominant sweet components. In the large-sized wild fish (W6), the contents of Gly and Ala reached 1.16 g/100 g and 1.27 g/100 g, respectively, which were significantly higher than those in the synchronous farmed group (F5: Gly 0.99 g/100 g; Ala 1.16 g/100 g) (*p* < 0.05).

The content of Bitter Amino Acids (BAA, including Val, Ile, Leu, Phe, His, Arg, and Met) provided the background complexity of the flavor. Although categorized as bitter, Arginine (Arg) is also a key contributor to the overall distinct seafood flavor (DAA). The quantitative analysis indicated that wild fish maintained higher levels of these specific amino acids (6.76–7.01 g/100 g in W2–W6) compared to the farmed groups (e.g., F5: 6.19 g/100 g) (*p* < 0.05).

The content of Delicious Amino Acids (DAAs, comprising Asp, Glu, Gly, Ala, and Arg) serves as a comprehensive index for seafood palatability. Consistent with the trend of total amino acids, the DAA content in farmed fish reached its maximum at 6 months (8.68 g/100 g) but declined significantly to 7.08 g/100 g at 12 months (*p* < 0.05). In contrast, wild fish maintained consistently higher DAA levels, with values remaining stable above 8.3 g/100 g in the W2–W6 groups. This quantitative advantage suggests that wild *P. major* possesses a richer substrate for flavor development compared to the farmed population.

### 3.4. Fatty Acid Profile

**Figure 5:**
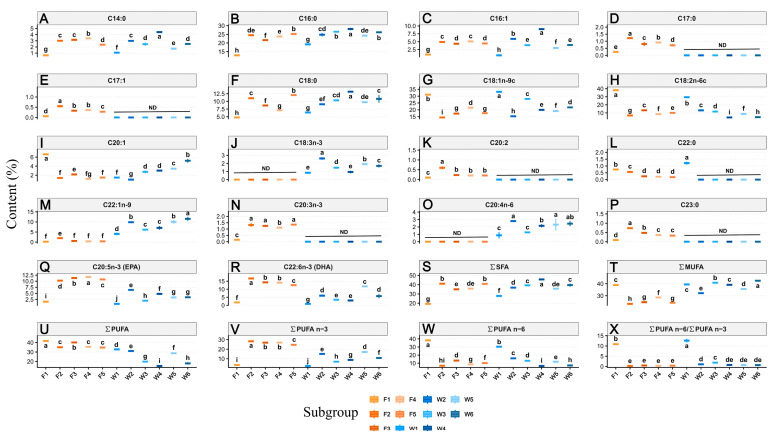
Fatty acid profiles of wild and farmed *P. major*. The panels display the relative percentage (%) of individual fatty acids (**A**–**T**) and summarized lipid categories (**U**–**X**). Specific panels include (**H**) Linoleic acid (C18:2n-6), (**M**) C22:1n-9, (**Q**) EPA (C20:5n-3), and (**R**) DHA (C22:6n-3). Panels (**U**–**X**) present the total PUFA, n-3 PUFA, n-6 PUFA, and the n-6/n-3 ratio, respectively. Data are expressed as mean ± SD. ND: <0.0033%. Different letters indicate significant differences (*p* < 0.05).

The fatty acid compositions of *P. major* muscle are presented in Table 7, with the specific signatures and ratios detailed in Figure 5. Significant variations were observed among the experimental groups (*p <* 0.05). Palmitic acid (C16:0) was the predominant saturated fatty acid (SFA) across all samples (12.84–28.10%), followed by stearic acid (C18:0). The total SFA content in farmed fish followed a clear temporal pattern: it was lowest at 3 months (F1, 19.32%) but increased rapidly to stabilize between 35.01% and 41.06% in the later stages (F2–F5). In contrast, the SFA content in wild fish showed a fluctuating upward trend with body size, reaching a peak of 45.68% in the W4 group.

Regarding monounsaturated fatty acids (MUFA), the wild population exhibited a distinct quantitative advantage. The total MUFA content in larger wild fish (W3–W6, 35.59–42.49%) was consistently higher than that in the size-matched farmed groups (F3–F5, 24.18–28.56%) (*p* < 0.05). Notably, C22:1n-9 was specifically enriched in the wild population, increasing with size to reach 11.52% in the W6 group. In comparison, this fatty acid was detected at negligible levels (<0.6%) in most farmed groups, with a minor exception in the F2 group (1.98%).

For polyunsaturated fatty acids (PUFA), the farmed group displayed a drastic shift in composition during the growth cycle. The juvenile F1 group exhibited the highest total PUFA content (41.69%), driven primarily by an exceptionally high level of linoleic acid (C18:2n-6c, 38.19%), which resulted in the highest n-6 PUFA content and an n-6/n-3 ratio of 10.90. However, from 6 months onwards (F2–F5), the fatty acid profile of farmed fish underwent a significant reversal: the level of C18:2n-6c decreased sharply (6.81–13.24%), while the content of n-3 PUFA increased markedly to a range of 24.63–28.26%. Specifically, the contents of eicosapentaenoic acid (EPA, C20:5n-3) and docosahexaenoic acid (DHA, C22:6n-3) in farmed fish (F2–F5) were maintained at high levels (>10% and >12%, respectively). Crucially, these values were significantly higher than those observed in all wild groups (*p <* 0.05), where EPA and DHA levels fluctuated considerably and generally remained lower (EPA: 0.69–6.43%; DHA: 0.89–11.78%).

Consequently, the ratio of ∑n-6/∑n-3 PUFA differed significantly among groups (*p <* 0.05). Although extremely high ratios (>10) were observed in the early juvenile stages of both populations (F1 and W1), the ratio in farmed fish dropped rapidly and remained at a nutritionally favorable low level (0.24–0.49) from F2 to F5. This was significantly lower than the ratios observed in wild fish of corresponding sizes (0.66–1.87), indicating that, with the exception of the juvenile stage (F1), farmed *P. major* accumulates a higher proportion of beneficial n-3 fatty acids.

### 3.5. Chromaticity Characteristics

**Figure 6:**
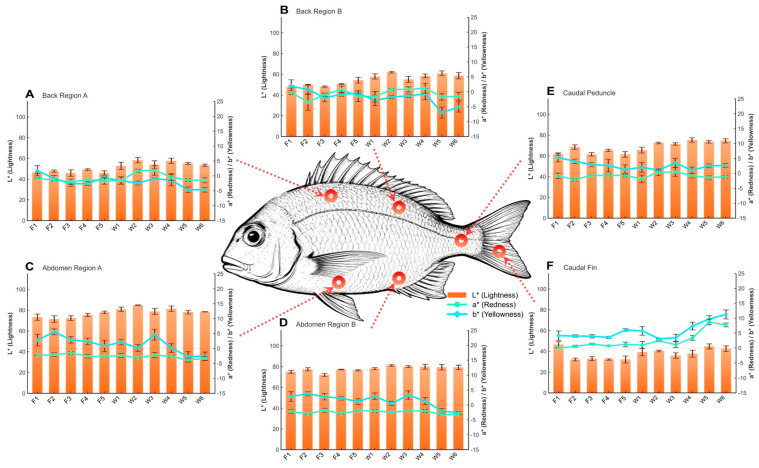
Spatiotemporal variations in body coloration and chromaticity characteristics. The central schematic illustrates the measurement points on the fish body. Bar charts (**A**–**F**) display the quantitative changes in L* (Lightness, orange bars), a* (Redness, green lines), and b* (Yellowness, blue lines) across growth stages. The results highlight the significant melanosis (lower L*) in farmed fish and the vivid reddish pigmentation (high a*) in the caudal fin of wild specimens (Panel (**F**)).

The chromaticity coordinates (L*, a*, b*) measured at different locations (back, abdomen, caudal peduncle, and caudal fin) of *P. major* are presented in Table 8, and the comparative analysis of these body color traits is visualized in Figure 6. Regarding lightness (L*), the wild population exhibited a significantly superior lustrous appearance compared to the farmed population (*p <* 0.05). Specifically, in the dorsal regions (Back Region A and Back Region B) and the caudal peduncle, the L* values of most wild groups were significantly higher than those of the size-matched farmed groups. For instance, the W2 group recorded the highest lightness in Back region B (61.95), which was significantly higher than the range observed in all farmed groups (47.80–54.33) (*p <* 0.05). Although the abdomen was naturally the brightest anatomical region for all fish, the wild specimens (e.g., W2 with an L* of 84.68) still maintained a significant quantitative advantage over the farmed counterparts (e.g., F2 with an L* of 71.39). These results indicate that the aquaculture environment led to a significant reduction in skin lightness, resulting in a darker visual appearance in farmed fish.

Redness (a*), a critical indicator for the visual quality of *P. major*, showed distinct variations primarily in the caudal fin. In the back and abdomen regions, a* values were mostly negative or near zero across all groups, indicating a greenish hue with minimal variation between populations. However, a striking contrast was observed in the caudal fin: the a* values in farmed fish (F1–F5) remained at a negligible level (0.04–1.29) and did not show significant improvement with age. In contrast, the a* values in the caudal fin of wild fish increased significantly with body size, peaking at 8.73 and 7.75 in the W5 and W6 groups, respectively. These values were significantly higher than those in all farmed groups (*p <* 0.05). This quantitative evidence highlights the vivid reddish pigmentation characteristic of the wild seabream tail, a trait that was notably absent in the farmed specimens.

The trend for yellowness (b*) paralleled that of redness, with the wild population displaying stronger pigment deposition in specific areas. In the caudal fin, the W6 group exhibited the highest b* value (11.28), which was significantly higher than that of the F5 group (6.07) (*p <* 0.05), reflecting a distinct golden hue in the tail of wild fish. Notably, in the dorsal regions (Back B), larger wild fish (W5 and W6) showed significant negative b* values (−6.98 and −5.09, respectively), tending towards blueness. This colorimetric shift is likely associated with the characteristic cobalt-blue spots often found on the back of wild *P. major*, a feature that was less pronounced or absent in farmed fish. Overall, wild *P. major* outperformed farmed fish in terms of lightness, as well as the redness and yellowness of the caudal fin.

## 4. Discussion

### 4.1. Variation in Proximate Composition Between Wild and Farmed Compositions

In the present study, structured as a size-class-based cross-sectional comparison, the proximate composition of *P. major* muscle was significantly influenced by the culture environment and body size. A strong inverse relationship between moisture and total lipid contents was observed, which is a well-documented phenomenon in various fish species [34,35,36]. The significantly higher lipid content in farmed fish compared to their wild counterparts can be primarily attributed to the imbalance between dietary energy intake and energy expenditure. Similar phenomena have been widely reported in other farmed fish species. While the literature frequently notes that farmed salmon exhibit higher fat content than wild populations due to high-energy feeds, wild salmonids can actually attain comparable somatic lipid levels during their open-ocean feeding phase; their characteristically low lipid content at capture stems from extensive lipid mobilization for gonadogenesis and spawning migration [37]. Conversely, for marine perciforms such as *P. major*, the continuous provision of nutrient-dense formulated diets in aquaculture fundamentally overrides natural energetic constraints, potentially contributing to consistently higher somatic lipid deposition than that achieved by wild counterparts. This diet-driven accumulation is mirrored in cage-farmed freshwater fish in Cambodia, which exhibit significantly higher lipid levels than wild populations due to the consumption of fat-rich industrial pellets and restricted physical activity [38]. In natural habitats, wild fish navigate complex environments with unpredictable food availability, requiring high energy expenditure for foraging, predator avoidance, and migration. Consequently, evolutionary physiology models confirm that fish facing constant environmental pressures maintain significantly leaner muscle tissue and lower lipid reserves compared to sedentary, cage-reared counterparts [39].

Furthermore, the confined space in sea cages restricts the physical activity of farmed fish, further promoting lipid deposition [40]. It is worth noting that the lipid content in the juvenile farmed stage (F1) was relatively low and similar to that of wild fish. This suggests that during the early rapid growth stage, metabolic energy is primarily allocated to somatic growth and protein synthesis rather than lipid storage. This phenomenon aligns with the ontogenetic energy allocation models described by Biro et al. [41], where juvenile fish allocate nearly all acquired energy to somatic growth to minimize size-dependent mortality. Furthermore, Post and Parkinson (2001) demonstrated that a somatic growth rate maximization strategy is evolutionarily optimal for young cohorts [42], delaying significant lipid accumulation until a critical body size is achieved.

Regarding crude protein, both wild and farmed fish exhibited high protein levels (>17%), confirming *P. major* as a protein-rich food source. The wild population generally maintained a stable and high protein content, which might be related to their continuous swimming activity. Studies indicate that the low-exercise environment in captivity shifts energy allocation towards lipid deposition, whereas wild fish maintain a leaner, protein-rich body composition to support higher swimming demands [43]. Sustained physical activity in nature activates the mTOR signaling pathway, which promotes protein synthesis and induces muscle fiber hypertrophy [44]. Although our results showed a lower protein content in farmed fish during early developmental stages, it gradually increased and reached levels comparable to wild fish in later stages. This increase in protein content aligns with the concept of chemical maturity described by Shearer [45], which posits that protein deposition is endogenously controlled and dependent on body size. Regarding ash content, values remained stable across all groups. This homeostatic capacity is supported by Baek and Cho [46], who observed that the whole-body ash content of *P. major* remained stable across all experimental groups. Notably, this stability persisted even though the dietary ash content increased significantly from 9.8% to 13.6% with the inclusion of tuna by-product meal. These findings suggest that mineral levels in *P. major* are strongly physiologically regulated and are relatively insensitive to dietary fluctuations.

First, due to the inherent logistical constraints in determining the absolute age of wild populations via otolith analysis, the comparison between farmed (age-based) and wild (size-based) cohorts relied on a body-weight matching strategy. The significant weight disparity in the largest cohorts (e.g., W6 vs. F5) precludes a perfect ontogenetic alignment, highlighting the need for future studies to incorporate covariate analyses (e.g., ANCOVA) based on precise age estimations. Secondly, although the farmed cohorts were reared under identical commercial management, stocking density, and feeding regimes to minimize environmental variance, potential ‘cage effects’ were not explicitly incorporated as a variable in the current statistical models, which should be addressed in future experimental designs. Similarly, for the wild populations, while all specimens were collected from a continuous overarching geographic region to minimize macro-environmental differences, potential micro-spatial ‘site effects’ from specific capture locations were also not isolated in our analysis.

### 4.2. Comparative Analysis of Amino Acid Profiles and Flavor Quality

Amino acids are widely recognized as pivotal indicators for evaluating the nutritional value and sensory characteristics of muscle foods [47]. In fish muscle, Jiang et al. [48] demonstrated that essential and flavor-contributing amino acids are critical determinants of flesh quality, directly influencing both nutritive value and umami/sweet taste profiles. Similar contributions of amino acids to sensory attributes have also been well-documented in other meat products, such as dry-cured ham [49], highlighting their universal importance in muscle food evaluation. In the present study, both farmed and wild *P. major* exhibited high lysine contents and EAA/TAA ratios that met the FAO/WHO standards for high-quality protein, confirming the species as a superior dietary protein source for humans. This conclusion aligns with recent metabolomic profiling of *P. major* [50], which characterized the species extensive amino acid composition and reaffirmed its status as a valuable source of aquatic protein rich in essential amino acids.

However, distinct accumulation patterns were observed between the two populations. Specifically, wild fish, particularly the larger individuals (groups W5–W6), demonstrated higher Essential Amino Acid Indexes (*EAAI*) compared to their farmed counterparts. This nutritional superiority can be largely attributed to the dietary diversity in the wild environment. This finding is consistent with Oztekin et al. [51], who reported similarly higher *EAAI* values in wild populations of the related seabream species *Pagellus acarne* compared to farmed groups, linking this advantage to the consumption of natural food resources. The natural prey of wild fish, such as crustaceans, mollusks, and small fish, provides a balanced amino acid profile that closely aligns with the somatic synthesis requirements. In contrast, while commercial feeds maintain consistent crude protein levels, the formulation of commercial feeds may partially limit the deposition efficiency of specific essential amino acids in muscle tissue. This nutritional divergence aligns with the observations of Wang et al. in *Cyprinus carpio haematopterus* [15], where wild populations possessed significantly higher ratios of essential to total amino acids (EAA/TAA). Although EAA/TAA ratios were relatively stable in the present study, the superior *EAAI* observed in wild *P. major* similarly indicates that the balanced amino acid profile provided by natural aquatic food webs facilitates more efficient high-quality protein deposition than commercial feeds.

In addition to the *EAAI*, the *F*-value (the molar ratio of branched-chain amino acids to aromatic amino acids) serves as a critical indicator for the functional quality of peptides. Studies by Mao et al. [52] and Wang et al. [53] have highlighted the significant physiological benefits of peptides with high *F*-values, particularly regarding hepatoprotection and the regulation of energy metabolism. Although the specific therapeutic formulations investigated in these studies typically require *F*-values exceeding 20, dietary muscle proteins naturally possessing an *F*-value above 2.0 are also generally recognized as having a favorable amino acid balance. In the present study, the *F*-values for all *P. major* groups ranged from 2.34 to 2.54. These results suggest that, regardless of culture environment, *P. major* muscle protein offers potential functional benefits. However, mirroring the *EAAI* trend, wild fish generally maintained slightly higher and more stable F-values (peaking at 2.54 in W4) compared to the late-stage farmed fish (2.34–2.37). This stability in wild populations likely reflects the consistent intake of high-quality animal protein from natural prey, whereas the slight fluctuation in farmed fish may be attributed to differences in dietary protein composition or metabolic responses to the intensive culture environment.

It is noteworthy that the amino acid content in farmed fish showed significant seasonal fluctuations, with a marked decrease in TAA and EAA levels observed at F4. This phenomenon is likely associated with metabolic adaptations to seasonal water temperature changes. According to Biswas et al. [54], low water temperatures can significantly reduce protein retention efficiency in *P. major* by suppressing metabolic rates and increasing the degradation of newly synthesized body protein, thereby limiting amino acid accumulation during colder periods. During periods of low temperature, feed intake in farmed fish typically declines, and protein synthesis is inhibited. Furthermore, to maintain basal metabolism under thermal stress, fish may mobilize specific muscle proteins or free amino acids as energy substrates, leading to a temporary depletion of the amino acid pool [55,56]. Specifically, metabolic studies indicate that thermal stress enhances amino acid catabolism and gluconeogenesis to meet elevated energy demands, often at the expense of somatic protein synthesis [56]. In contrast, wild fish, potentially influenced by the need for continuous high-intensity swimming for foraging and migration, possess more robust metabolic functions. This active lifestyle promotes metabolic adaptations that allow them to maintain relatively stable amino acid accumulation even in later growth stages [15].

Free amino acids are widely recognized as the key determinants of fish flavor. Protein degradation products, particularly free amino acids, are the primary sources of umami and sweetness [57]. Our results indicated that the flavor-related amino acid profile in wild fish was chemically richer than that of the farmed group, characterized by significantly higher levels of Delicious Amino Acids (DAA). This quantitative advantage was driven by the enrichment of specific functional groups, specifically Umami Amino Acids (UAA) and Sweet Amino Acids (SAA). Specifically, the higher content of glutamic acid (UAA) and the sweet amino acids glycine and alanine (SAA) in large-sized wild fish suggests a potential for a more intense richer biochemical foundation for these sensory attributes. Additionally, although arginine is chemically classified as a Bitter Amino Acid (BAA) due to its intrinsic taste, it is simultaneously a key component of DAA. In the complex food matrix, arginine acts synergistically with other amino acids to enhance the overall complexity of the potential flavor profile. The observed accumulation of these flavor-active compounds in wild fish is not only related to the diet–flavor transfer mechanism but may also be linked to exercise physiology. For instance, Jia et al. [58] recently demonstrated in the large yellow croaker (*Larimichthys crocea*) that swimming exercise significantly enhances muscle umami attributes by upregulating key flavor amino acids such as alanine and glutamic acid, confirming that physical activity is a critical driver of flesh flavor formation independent of dietary sources. The rigorous physical activity of wild fish in natural waters may stimulate the compensatory accumulation of these non-essential amino acids in muscle tissue, thereby contributing to the richer flavor substrate characteristic of wild *P. major*. However, it is important to note that the present study measured total hydrolyzed amino acids rather than free amino acids (FAAs). While free amino acids are the primary drivers of immediate taste perception, the total amino acid profile analyzed herein represents the overarching biochemical foundation of the muscle. These protein-bound amino acids act as crucial precursors that can be released through enzymatic degradation during post-mortem aging or thermal hydrolysis during cooking, thereby ultimately contributing to the final flavor profile of the fish.

### 4.3. Dietary Imprinting on Fatty Acid Profiles and Nutritional Evaluation

It is widely accepted that the fatty acid composition of fish muscle closely mirrors dietary lipid profiles [59]. In the present study, farmed and wild *P. major* exhibited distinct metabolic signatures. Notably, the juvenile farmed stage (F1) displayed markedly elevated concentrations of linoleic acid (C18:2n-6c, >38%) and total n-6 polyunsaturated fatty acids (PUFAs), resulting in an n-6/n-3 ratio that far exceeded recommended values. This accumulation pattern likely reflects the lipid composition of the diet consumed during the early growth stage. This conclusion is supported by an extensive body of literature demonstrating that the inclusion of dietary vegetable oils significantly elevates muscle linoleic acid levels and total n-6 PUFA content in farmed fish [60]. Since marine fish lack sufficient enzymatic activity to convert C18:2n-6c into long-chain PUFAs, these exogenous fatty acids are deposited directly into the muscle tissue, effectively mirroring the dietary lipid profile [61]. However, as the culture period progressed (F2–F5), the fatty acid profile of farmed fish underwent a marked transition. Given that marine teleosts lack sufficient endogenous capacity to synthesize long-chain polyunsaturated fatty acids from C18 precursors, this specific decline in C18:2n-6c and rapid deposition of EPA and DHA is not driven by an endogenous metabolic turnover, but rather strongly suggests a shift in the dietary lipid profile. This reversal is consistent with the wash-out effect, indicating a dietary shift towards lipid sources rich in marine-derived n-3 LC-PUFA [62]. Such dietary modulation enables farmed seabream to effectively enrich their muscle tissue with n-3 LC-PUFAs, ensuring a nutritional profile that is pivotal for human health [9]. Although fatty acids were evaluated as relative percentages, the significantly higher total lipid content in farmed fish inherently provides greater absolute amounts of EPA and DHA per edible portion. The overall fatty acid profile of the farmed cohorts reflects the characteristic lipid accumulation patterns associated with standard commercial rearing environments.

Notably, the exceptionally high level of C22:1n-9 (>10%) was observed in wild fish, particularly in larger individuals (W5-W6), whereas this fatty acid was negligible in the farmed group. High concentrations of C22:1n-9 are recognized as a specific trophic marker originating from marine zooplankton (e.g., copepods) rich in wax esters [63]. While C22:1n-9 can be introduced into aquaculture via specific fish oil inclusions, its exceptionally high accumulation (>10%) in wild specimens—compared to its negligible detection in the farmed cohorts—suggests it may serve as a promising candidate biochemical indicator for wild-caught *P. major* in this specific region. However, broader validation across multiple seasons and geographic origins is required before establishing it as a robust authentication marker. Furthermore, the elevated monounsaturated fatty acid (MUFA) content in wild fish likely reflects their natural diet, which is inherently rich in these compounds [64]. Furthermore, the unexpectedly lower relative percentages of EPA and DHA in wild specimens, despite their marine origin, likely result from a combination of variable prey resources and the disproportionate enrichment of other specific fatty acids (such as C22:1n-9), which mathematically dilutes the relative fraction of n-3 LC-PUFAs. This pattern aligns with an energy allocation strategy optimized for fluctuating environmental conditions [9], wherein wild fish prioritize lean mass and metabolic flexibility over lipid storage.

Furthermore, because commercial and natural diets were not quantitatively analyzed in parallel, the specific contribution of dietary lipid profiles to the observed muscle fatty acid variations remains fundamentally deductive.

### 4.4. Mechanisms Underlying Coloration Differences

Body color is a primary sensory attribute determining the commercial value of *P. major*, with consumers showing a strong preference for individuals possessing vivid reddish pigmentation [65]. In the present study, wild *P. major* exhibited significantly superior redness (a*) and yellowness (b*), particularly in the caudal fin, which directly reflects their ability to acquire carotenoids through the natural food web [66]. Since fish cannot synthesize carotenoids de novo, wild sea bream acquire their vivid coloration by chronically feeding on marine crustaceans (e.g., shrimp and crabs) rich in natural pigments like astaxanthin, canthaxanthin, and lutein, which subsequently deposit in their chromatophores [67]. In contrast, the farmed fish exhibited noticeable skin darkening. Because feed composition was not analyzed in this study, it is difficult to confirm whether this darkening resulted from a lack of dietary carotenoids, or if environmental stress in cage culture impaired the pigment deposition process [68].

Additionally, a significant difference was observed in lightness (L*). Farmed fish showed significantly lower L* values than wild fish, presenting a darker or dull appearance, a condition often referred to as melanosis or the darkening phenomenon in aquaculture [69]. This phenomenon is typically associated with the proliferation of melanophores or the abnormal dispersion of melanin. Factors in the aquaculture environment, such as high stocking density, altered light exposure due to sea cages, and crowding stress, may activate the hypothalamic–pituitary axis to secrete melanocyte-stimulating hormone (MSH), thereby increasing melanin deposition and masking the underlying red pigmentation [70,71,72]. Furthermore, our study found that large-sized wild fish exhibited negative b* values (tending towards blueness) on the dorsal side, corresponding to the characteristic scattered cobalt-colored patches on the upper half of the body. This unique blue color is a structural color generated by light reflection from iridophores in the dermis and is a typical signature of high-quality wild seabream [65]. The absence or fading of this blue feature in farmed fish suggests a potential lack of specific micronutrients or alterations in the arrangement of iridophores caused by the farming environment [73,74].

## 5. Conclusions

This study presents a size-class-based cross-sectional comparison of the biochemical and chromatic profiles between wild and cage-farmed *P. major*. The results indicate that farmed cohorts exhibited greater somatic lipid deposition and higher relative levels of n-3 LC-PUFAs, while wild specimens showed higher concentrations of specific protein-bound amino acids and more pronounced caudal fin redness. These phenotypic baselines suggest potential associations between the rearing environment and the observed nutritional and chromatic divergences. However, because variables such as precise dietary intake, physical activity, and pigment metabolism were not directly measured, definitive causal relationships cannot be established. Furthermore, as flavor perception relies on unmeasured free amino acids and volatile compounds, the amino acid profiles presented here reflect only a potential biochemical foundation rather than direct sensory superiority. Future research incorporating longitudinal age tracking, direct dietary analysis, and comprehensive sensory evaluation is necessary to fully elucidate the underlying mechanisms.

## Figures and Tables

**Table 1:** Conventional nutrient composition of wild and net-pen-cultured *P. major* muscle of various sizes (%, wet weight).

Group/Index	Crude Protein	Total Lipid	Moisture	Total Ash
F1	17.80 ± 0.53 ^e^	1.20 ± 0.17 ^cd^	79.23 ± 0.42 ^a^	1.60 ± 0.17 ^a^
F2	19.90 ± 0.17 ^d^	2.3 ± 0.44 ^b^	75.87 ± 0.40 ^cd^	1.87 ± 0.64 ^a^
F3	20.17 ± 0.85 ^bcd^	2.90 ± 0.30 ^b^	74.60 ± 1.56 ^ef^	1.93 ± 0.93 ^a^
F4	20.10 ± 0.44 ^cd^	4.77 ± 0.29 ^a^	73.33 ± 0.50 ^g^	1.63 ± 0.06 ^a^
F5	20.87 ± 0.55 ^ab^	4.53 ± 1.06 ^a^	73.17 ± 0.60 ^g^	1.43 ± 0.15 ^a^
W1	19.77 ± 0.06 ^d^	1.20 ± 0.10 ^cd^	77.00 ± 0.10 ^b^	1.57 ± 0.06 ^a^
W2	20.73 ± 0.15 ^abc^	1.63 ± 0.06 ^c^	75.53 ± 0.25 ^de^	1.77 ± 0.06 ^a^
W3	20.17 ± 0.12 ^bcd^	2.43 ± 0.06 ^b^	75.33 ± 0.45 ^def^	1.60 ± 0.00 ^a^
W4	21.03 ± 0.15 ^a^	2.37 ± 0.06 ^b^	74.33 ± 0.15 ^f^	1.87 ± 0.06 ^a^
W5	20.07 ± 0.15 ^cd^	0.87 ± 0.06 ^d^	76.83 ± 0.12 ^bc^	1.63 ± 0.06 ^a^
W6	20.17 ± 0.12 ^bcd^	1.13 ± 0.06 ^cd^	76.73 ± 0.25 ^bc^	1.67 ± 0.06 ^a^

Note: Data are expressed as mean ± standard deviation (SD). Values for the same parameter with different superscript letters indicate significant differences (*p* < 0.05).

**Table 2:** Amino acid composition and content in muscle of wild and cage-cultured *P. major* (%, wet weight).

Amino Acids	F1	F2	F3	F4	F5	W1	W2	W3	W4	W5	W6
^#^ Lys	1.76 ± 0.17 ^cd^	2.02 ± 0.09 ^a^	1.78 ± 0.17 ^bcd^	1.64 ± 0.01 ^d^	1.72 ± 0.17 ^cd^	1.83 ± 0.03 ^abcd^	1.99 ± 0.01 ^ab^	2.01 ± 0.01 ^a^	1.98 ± 0.01 ^ab^	2.00 ± 0.03 ^a^	1.93 ± 0.01 ^abc^
Ser	0.76 ± 0.06 ^de^	0.84 ± 0.03 ^abc^	0.80 ± 0.04 ^cd^	0.70 ± 0.01 ^f^	0.74 ± 0.06 ^ef^	0.81 ± 0.03 ^cd^	0.89 ± 0.00 ^a^	0.86 ± 0.01 ^ab^	0.84 ± 0.01 ^abc^	0.82 ± 0.01 ^bc^	0.82 ± 0.01 ^bc^
Asp	1.87 ± 0.16 ^cd^	2.11 ± 0.09 ^a^	1.89 ± 0.20 ^bcd^	1.73 ± 0.02 ^d^	1.83 ± 0.19 ^d^	1.90 ± 0.05 ^bcd^	2.07 ± 0.01 ^ab^	2.13 ± 0.01 ^a^	2.11 ± 0.01 ^a^	2.04 ± 0.01 ^abc^	2.03 ± 0.01 ^abc^
^#^ Thr	0.86 ± 0.07 ^bcd^	0.96 ± 0.03 ^a^	0.87 ± 0.09 ^bcd^	0.80 ± 0.02 ^d^	0.85 ± 0.09 ^cd^	0.84 ± 0.05 ^cd^	0.92 ± 0.01 ^ab^	0.99 ± 0.01 ^a^	0.97 ± 0.01 ^a^	0.94 ± 0.01 ^ab^	0.93 ± 0.01 ^abc^
^#^ Trp	0.14 ± 0.01 ^f^	0.16 ± 0.01 ^cd^	0.16 ± 0.01 ^bc^	0.17 ± 0.01 ^ab^	0.17 ± 0.01 ^a^	0.15 ± 0.00 ^e^	0.16 ± 0.00 ^bcd^	0.15 ± 0.00 ^d^	0.15 ± 0.00 ^e^	0.16 ± 0.00 ^bcd^	0.14 ± 0.00 ^f^
Ala	1.19 ± 0.10 ^bcd^	1.27 ± 0.06 ^ab^	1.24 ± 0.04 ^abc^	1.07 ± 0.02 ^e^	1.16 ± 0.05 ^cde^	1.11 ± 0.10 ^de^	1.28 ± 0.04 ^ab^	1.28 ± 0.01 ^ab^	1.29 ± 0.00 ^a^	1.20 ± 0.01 ^abcd^	1.27 ± 0.01 ^ab^
Glu	2.68 ± 0.21 ^cd^	3.07 ± 0.12 ^a^	2.76 ± 0.28 ^bcd^	2.39 ± 0.07 ^e^	2.53 ± 0.26 ^de^	2.94 ± 0.13 ^abc^	3.15 ± 0.01 ^a^	3.00 ± 0.02 ^ab^	2.91 ± 0.03 ^abc^	2.92 ± 0.02 ^abc^	2.92 ± 0.03 ^abc^
Pro	0.69 ± 0.04 ^bc^	0.71 ± 0.06 ^abc^	0.76 ± 0.12 ^ab^	0.59 ± 0.05 ^d^	0.67 ± 0.07 ^bcd^	0.67 ± 0.04 ^bcd^	0.80 ± 0.02 ^a^	0.66 ± 0.00 ^cd^	0.68 ± 0.01 ^bcd^	0.63 ± 0.02 ^cd^	0.72 ± 0.01 ^abc^
Gly	0.92 ± 0.06 ^ab^	0.97 ± 0.06 ^ab^	1.16 ± 0.37 ^a^	0.85 ± 0.05 ^b^	0.99 ± 0.13 ^ab^	0.89 ± 0.04 ^b^	1.02 ± 0.01 ^ab^	1.01 ± 0.01 ^ab^	1.06 ± 0.00 ^ab^	0.93 ± 0.00 ^ab^	1.16 ± 0.00 ^a^
^#^ Val	0.91 ± 0.08 ^d^	1.03 ± 0.05 ^a^	0.90 ± 0.11 ^d^	0.86 ± 0.01 ^d^	0.93 ± 0.10 ^bcd^	0.92 ± 0.01 ^cd^	1.01 ± 0.01 ^abc^	1.03 ± 0.01 ^ab^	1.04 ± 0.00 ^a^	1.02 ± 0.01 ^abc^	1.03 ± 0.01 ^a^
^#^ Ile	0.83 ± 0.08 ^cd^	0.99 ± 0.04 ^a^	0.84 ± 0.13 ^cd^	0.78 ± 0.04 ^d^	0.85 ± 0.10 ^bcd^	0.89 ± 0.05 ^abcd^	0.92 ± 0.02 ^abc^	0.98 ± 0.01 ^a^	0.98 ± 0.00 ^a^	0.96 ± 0.01 ^ab^	0.96 ± 0.01 ^ab^
^#^ Leu	1.51 ± 0.13 ^bcd^	1.72 ± 0.08 ^a^	1.51 ± 0.21 ^bcd^	1.38 ± 0.06 ^d^	1.48 ± 0.17 ^cd^	1.59 ± 0.11 ^abc^	1.69 ± 0.02 ^ab^	1.73 ± 0.01 ^a^	1.71 ± 0.01 ^a^	1.66 ± 0.02 ^abc^	1.63 ± 0.01 ^abc^
Tyr	0.63 ± 0.06 ^b^	0.67 ± 0.05 ^ab^	0.58 ± 0.08 ^b^	0.58 ± 0.02 ^b^	0.62 ± 0.09 ^b^	0.64 ± 0.11 ^b^	0.67 ± 0.01 ^ab^	0.67 ± 0.01 ^ab^	0.63 ± 0.01 ^b^	0.75 ± 0.01 ^a^	0.67 ± 0.00 ^ab^
^#^ Phe	0.76 ± 0.07 ^ab^	0.84 ± 0.04 ^a^	0.76 ± 0.09 ^ab^	0.71 ± 0.02 ^a^	0.76 ± 0.08 ^ab^	0.79 ± 0.16 ^ab^	0.78 ± 0.01 ^ab^	0.82 ± 0.01 ^ab^	0.84 ± 0.01 ^ab^	0.75 ± 0.02 ^ab^	0.77 ± 0.02 ^ab^
His	0.48 ± 0.04 ^d^	0.53 ± 0.02 ^bcd^	0.48 ± 0.08 ^d^	0.48 ± 0.02 ^d^	0.50 ± 0.05 ^cd^	0.53 ± 0.04 ^bcd^	0.61 ± 0.00 ^a^	0.55 ± 0.01 ^abc^	0.56 ± 0.00 ^abc^	0.58 ± 0.07 ^ab^	0.52 ± 0.01 ^bcd^
Arg	1.13 ± 0.11 ^d^	1.26 ± 0.06 ^abc^	1.22 ± 0.03 ^bc^	1.05 ± 0.02 ^e^	1.13 ± 0.06 ^d^	1.20 ± 0.01 ^cd^	1.31 ± 0.01 ^a^	1.30 ± 0.01 ^ab^	1.29 ± 0.01 ^ab^	1.25 ± 0.01 ^abc^	1.30 ± 0.01 ^ab^
^#^ Met	0.56 ± 0.05 ^abcd^	0.54 ± 0.05 ^bcd^	0.51 ± 0.06 ^d^	0.52 ± 0.01 ^cd^	0.53 ± 0.08 ^bcd^	0.50 ± 0.01 ^d^	0.57 ± 0.01 ^abcd^	0.60 ± 0.01 ^ab^	0.58 ± 0.01 ^abc^	0.63 ± 0.00 ^a^	0.54 ± 0.01 ^bcd^
Cys	0.19 ± 0.02 ^bc^	0.19 ± 0.02 ^bc^	0.16 ± 0.02 ^cde^	0.17 ± 0.02 ^cd^	0.14 ± 0.05 ^de^	0.12 ± 0.01 ^e^	0.13 ± 0.01 ^e^	0.17 ± 0.01 ^cd^	0.17 ± 0.00 ^cd^	0.26 ± 0.01 ^a^	0.22 ± 0.01 ^b^
TAA	17.86 ± 1.47 ^c^	19.88 ± 0.90 ^a^	18.38 ± 1.18 ^bc^	16.46 ± 0.28 ^d^	17.62 ± 1.35 ^cd^	18.31 ± 0.35 ^bc^	19.96 ± 0.15 ^a^	19.95 ± 0.11 ^a^	19.79 ± 0.08 ^a^	19.48 ± 0.04 ^ab^	19.55 ± 0.09 ^ab^
EAA	7.33 ± 0.65 ^bc^	8.27 ± 0.36 ^a^	7.33 ± 0.96 ^bc^	6.86 ± 0.13 ^c^	7.30 ± 0.78 ^bc^	7.50 ± 0.35 ^abc^	8.03 ± 0.07 ^ab^	8.30 ± 0.04 ^a^	8.25 ± 0.24 ^ab^	8.11 ± 0.04 ^ab^	7.94 ± 0.05 ^ab^
NEAA	10.53 ± 0.82 ^de^	11.61 ± 0.55 ^ab^	11.05 ± 0.32 ^bcd^	9.60 ± 0.16 ^f^	10.32 ± 0.63 ^e^	10.81 ± 0.05 ^cde^	11.92 ± 0.07 ^a^	11.64 ± 0.06 ^ab^	11.54 ± 0.06 ^ab^	11.37 ± 0.05 ^abc^	11.61 ± 0.05 ^ab^
EAA/TAA	0.41 ± 0.00 ^a^	0.42 ± 0.00 ^a^	0.40 ± 0.03 ^a^	0.42 ± 0.00 ^a^	0.41 ± 0.02 ^a^	0.41 ± 0.02 ^a^	0.40 ± 0.00 ^a^	0.42 ± 0.00 ^a^	0.42 ± 0.00 ^a^	0.42 ± 0.00 ^a^	0.41 ± 0.00 ^a^
EAA/NEAA	0.70 ± 0.01 ^a^	0.71 ± 0.00 ^a^	0.66 ± 0.08 ^a^	0.71 ± 0.00 ^a^	0.71 ± 0.05 ^a^	0.69 ± 0.03 ^a^	0.67 ± 0.00 ^a^	0.71 ± 0.00 ^a^	0.72 ± 0.00 ^a^	0.71 ± 0.01 ^a^	0.68 ± 0.00 ^a^

Note: ^#^ Essential amino acid; TAA, total amino acids; EAA, essential amino acid; NEAA, non-essential amino acid. Value = mean ± SD. Values in the same row with different superscript letters indicate significant differences (*p* < 0.05).

**Table 3:** Essential amino acid composition (mg/g N) and nutritional evaluation indices (*EAAI*, *F*-value) of *P. major* muscle protein.

Amino Acids	F1	F2	F3	F4	F5	W1	W2	W3	W4	W5	W6
Lys	617.34 ± 44.59	635.65 ± 33.80	552.60 ± 99.04	510.14 ± 13.52	516.03 ± 47.56	578.63 ± 9.62	599.89 ± 3.35	625.04 ± 8.19	589.36 ± 2.72	622.97 ± 11.75	599.18 ± 1.64
Thr	301.77 ± 18.77	302.63 ± 12.77	271.39 ± 34.99	247.78 ± 5.99	253.52 ± 23.80	266.68 ± 14.98	276.32 ± 1.69	306.31 ± 4.05	287.25 ± 2.45	291.74 ± 0.78	287.19 ± 1.72
Trp	47.98 ± 0.69	49.20 ± 1.39	50.65 ± 1.69	51.82 ± 1.46	51.95 ± 2.60	46.59 ± 0.92	48.53 ± 0.38	48.02 ± 0.79	43.77 ± 0.14	49.11 ± 0.21	44.01 ± 0.16
Val	318.14 ± 22.49	323.59 ± 17.15	279.72 ± 39.43	269.59 ± 7.54	278.37 ± 27.92	290.90 ± 3.27	305.47 ± 0.83	318.73 ± 4.14	309.04 ± 2.24	316.66 ± 0.92	320.25 ± 0.04
Ile	292.40 ± 23.58	309.97 ± 15.29	260.19 ± 45.88	242.56 ± 10.70	254.44 ± 28.96	280.38 ± 15.45	276.33 ± 4.00	303.21 ± 4.02	291.21 ± 2.11	297.97 ± 0.82	296.49 ± 1.75
Leu	531.04 ± 36.61	541.40 ± 27.93	470.60 ± 77.59	428.12 ± 18.65	444.11 ± 47.92	501.76 ± 35.33	509.44 ± 4.57	536.04 ± 5.77	509.12 ± 2.15	517.02 ± 2.47	506.20 ± 1.11
Met + Cys	261.97 ± 17.50	230.45 ± 23.45	207.00 ± 26.01	215.75 ± 11.01	201.38 ± 16.30	197.08 ± 3.08	209.02 ± 3.08	239.05 ± 4.49	223.85 ± 0.56	274.09 ± 2.35	232.44 ± 1.33
Phe + Tyr	486.56 ± 33.27	474.41 ± 28.80	416.25 ± 56.08	401.23 ± 10.03	412.04 ± 43.65	451.26 ± 84.54	439.12 ± 3.38	464.64 ± 6.67	436.81 ± 0.62	467.20 ± 0.77	447.31 ± 2.46
Total	2857.19 ± 195.74	2867.30 ± 154.62	2508.40 ± 377.60	2367.00 ± 64.31	2411.85 ± 235.59	2613.28 ± 150.42	2664.12 ± 16.87	2841.04 ± 36.91	2690.43 ± 11.33	2836.74 ± 13.49	2733.07 ± 5.86
*EAAI*	85.22 ± 5.32	85.18 ± 3.96	75.89 ± 9.96	72.75 ± 1.95	73.75 ± 6.58	77.36 ± 3.58	79.35 ± 0.45	84.58 ± 1.15	79.80 ± 0.28	85.14 ± 0.34	81.11 ± 0.16
*F*	2.35 ± 0.01	2.48 ± 0.05	2.42 ± 0.08	2.34 ± 0.04	2.37 ± 0.03	2.42 ± 0.31	2.49 ± 0.02	2.49 ± 0.02	2.54 ± 0.01	2.42 ± 0.01	2.51 ± 0.02

Note: Data are expressed as mean ± SD. *EAAI*: Essential Amino Acid Index; *F*-value: Fisher ratio.

**Table 4:** Amino acid scores (AAS) of muscle in wild and net-pen-cultured *P. major.*

Amino Acids	F1	F2	F3	F4	F5	W1	W2	W3	W4	W5	W6
Lys	1.82 ± 0.13	1.87 ± 0.10	1.63 ± 0.29	1.50 ± 0.04	1.52 ± 0.14	1.70 ± 1.76	1.76 ± 0.01	1.84 ± 0.02	1.73 ± 0.01	1.83 ± 0.03	1.76 ± 0.00
Thr	1.21 ± 0.08	1.21 ± 0.05	1.09 ± 0.14	0.99 ± 0.02	1.01 ± 0.10	1.07 ± 0.06	1.11 ± 0.01	1.23 ± 0.02	1.15 ± 0.01	1.17 ± 0.00	1.15 ± 0.01
Trp	0.80 ± 0.01	0.82 ± 0.02	0.84 ± 0.03	0.86 ± 0.02	0.86 ± 0.04	0.78 ± 0.02	0.81 ± 0.01	0.80 ± 0.01	0.73 ± 0.00	0.82 ± 0.00	0.73 ± 0.00
Val	1.03 ± 0.07	1.04 ± 0.06	0.90 ± 0.13	0.87 ± 0.02	0.90 ± 0.09	0.94 ± 0.01	0.99 ± 0.00	1.03 ± 0.01	1.00 ± 0.01	1.02 ± 0.00	1.03 ± 0.00
Ile	1.17 ± 0.09	1.24 ± 0.06	1.04 ± 0.18	0.97 ± 0.04	1.02 ± 0.12	1.12 ± 0.06	1.11 ± 0.02	1.21 ± 0.02	1.16 ± 0.01	1.19 ± 0.00	1.19 ± 0.01
Leu	1.21 ± 0.08	1.23 ± 0.06	1.07 ± 0.18	0.97 ± 0.04	1.01 ± 0.11	1.14 ± 0.08	1.16 ± 0.01	1.22 ± 0.01	1.16 ± 0.00	1.18 ± 0.01	1.15 ± 0.00
Met + Cys	1.19 ± 0.08	1.05 ± 0.11	0.94 ± 0.12	0.98 ± 0.05	0.92 ± 0.07	0.90 ± 0.01	0.95 ± 0.01	1.09 ± 0.02	1.02 ± 0.00	1.25 ± 0.01	1.05 ± 0.01
Phe + Tyr	1.28 ± 0.09	1.25 ± 0.08	1.10 ± 0.15	1.06 ± 0.03	1.08 ± 0.11	1.19 ± 0.22	1.16 ± 0.01	1.22 ± 0.02	1.15 ± 0.00	1.23 ± 0.00	1.17 ± 0.01

**Table 5:** Chemical scores (CS) of muscle in wild and net-pen-cultured *P. major.*

Amino Acids	F1	F2	F3	F4	F5	W1	W2	W3	W4	W5	W6
Lys	1.40 ± 0.10	1.44 ± 0.08	1.25 ± 0.22	1.16 ± 0.03	1.17 ± 0.11	1.31 ± 0.02	1.36 ± 0.01	1.42 ± 0.02	1.34 ± 0.01	1.41 ± 0.03	1.36 ± 0.00
Thr	1.03 ± 0.06	1.04 ± 0.04	0.93 ± 0.12	0.85 ± 0.02	0.87 ± 0.08	0.91 ± 0.05	0.95 ± 0.01	1.05 ± 0.01	0.98 ± 0.01	1.00 ± 0.00	0.98 ± 0.01
Trp	0.48 ± 0.01	0.50 ± 0.01	0.51 ± 0.02	0.52 ± 0.01	0.52 ± 0.03	0.47 ± 0.01	0.49 ± 0.00	0.49 ± 0.01	0.44 ± 0.00	0.50 ± 0.00	0.44 ± 0.00
Val	0.72 ± 0.05	0.73 ± 0.04	0.63 ± 0.09	0.61 ± 0.02	0.63 ± 0.07	0.66 ± 0.01	0.69 ± 0.00	0.72 ± 0.01	0.70 ± 0.01	0.77 ± 0.00	0.78 ± 0.00
Ile	0.88 ± 0.07	0.94 ± 0.05	0.79 ± 0.14	0.73 ± 0.03	0.77 ± 0.09	0.85 ± 0.05	0.83 ± 0.01	0.92 ± 0.01	0.88 ± 0.01	0.90 ± 0.00	0.90 ± 0.01
Leu	1.00 ± 0.07	1.01 ± 0.05	0.88 ± 0.15	0.80 ± 0.03	0.83 ± 0.09	0.94 ± 0.07	0.95 ± 0.01	1.00 ± 0.01	0.95 ± 0.00	0.97 ± 0.00	0.95 ± 0.00
Met + Cys	0.68 ± 0.05	0.60 ± 0.06	0.54 ± 0.07	0.56 ± 0.03	0.52 ± 0.04	0.51 ± 0.01	0.54 ± 0.01	0.62 ± 0.01	0.58 ± 0.00	0.71 ± 0.01	0.60 ± 0.00
Phe + Tyr	0.86 ± 0.06	0.84 ± 0.05	0.74 ± 0.10	0.71 ± 0.02	0.73 ± 0.08	0.80 ± 0.15	0.78 ± 0.01	0.82 ± 0.01	0.77 ± 0.00	0.83 ± 0.00	0.79 ± 0.00

**Table 6:** Flavor-related amino acid composition (g/100 g fresh weight) in the muscle of wild and farmed *P. major.*

Amino Acids	F1	F2	F3	F4	F5	W1	W2	W3	W4	W5	W6
UAA	4.55 ± 0.37 ^cd^	5.18 ± 0.20 ^a^	4.66 ± 0.49 ^bcd^	4.12 ± 0.09 ^e^	4.37 ± 0.44 ^de^	4.84 ± 0.11 ^abc^	5.22 ± 0.02 ^a^	5.13 ± 0.03 ^a^	5.01 ± 0.04 ^ab^	4.96 ± 0.03 ^abc^	4.94 ± 0.03 ^abc^
SAA	3.73 ± 0.27 ^c^	4.04 ± 0.19 ^ab^	4.07 ± 0.29 ^ab^	3.41 ± 0.09 ^d^	3.74 ± 0.14 ^c^	3.65 ± 0.12 ^cd^	4.11 ± 0.05 ^ab^	4.14 ± 0.03 ^ab^	4.16 ± 0.02 ^ab^	3.89 ± 0.02 ^bc^	4.18 ± 0.03 ^a^
BAA	6.18 ± 0.55 ^bc^	6.92 ± 0.32 ^a^	6.21 ± 0.69 ^bc^	5.78 ± 0.12 ^c^	6.19 ± 0.63 ^bc^	6.41 ± 0.26 ^abc^	6.89 ± 0.07 ^a^	7.00 ± 0.04 ^a^	7.01 ± 0.02 ^a^	6.84 ± 0.02 ^ab^	6.76 ± 0.05 ^ab^
DAA	7.79 ± 0.62 ^d^	8.68 ± 0.38 ^ab^	8.28 ± 0.19 ^bc^	7.08 ± 0.12 ^e^	7.65 ± 0.47 ^d^	8.04 ± 0.01 ^cd^	8.83 ± 0.06 ^a^	8.72 ± 0.05 ^ab^	8.66 ± 0.04 ^ab^	8.35 ± 0.03 ^abc^	8.68 ± 0.05 ^ab^

Note: Data are expressed as mean ± SD. Different superscripts letters indicate significant differences (*p* < 0.05). Umami amino acid: Asp and Glu. Sweet amino acid: Thr, Ala, Gly and Ser. Bitter amino acid: Ile, Leu, Met, Phe, Val, His and Arg. Delicious amino acid: Asp, Arg, Glu, Gly and Ala.

**Table 7:** Fatty acid composition of muscle in wild and net-pen-cultured *P. major* (%, wet weight).

Fatty Acids	F1	F2	F3	F4	F5	W1	W2	W3	W4	W5	W6
C14: 0	0.64 ± 0.00 ^g^	3.00 ± 0.15 ^c^	3.16 ± 0.06 ^c^	3.40 ± 0.02 ^b^	2.36 ± 0.02 ^d^	1.07 ± 0.08 ^f^	2.99 ± 0.16 ^c^	2.45 ± 0.26 ^d^	4.41 ± 0.12 ^a^	1.72 ± 0.18 ^e^	2.49 ± 0.08 ^d^
C16: 0	12.84 ± 0.01 ^h^	24.49 ± 0.06 ^de^	21.64 ± 0.25 ^f^	23.80 ± 0.10 ^e^	25.25 ± 0.07 ^b^	19.19 ± 0.18 ^g^	24.80 ± 0.98 ^cd^	26.52 ± 0.12 ^b^	28.10 ± 0.70 ^a^	24.23 ± 0.17 ^de^	26.22 ± 0.40 ^b^
C16: 1	0.93 ± 0.00 ^g^	4.91 ± 0.10 ^c^	4.35 ± 0.08 ^d^	5.09 ± 0.03 ^c^	4.45 ± 0.01 ^d^	0.68 ± 0.07 ^h^	5.90 ± 0.03 ^b^	3.90 ± 0.13 ^e^	8.99 ± 0.06 ^a^	3.01 ± 0.21 ^f^	3.95 ± 0.24 ^e^
C17: 0	0.24 ± 0.01 ^e^	1.22 ± 0.03 ^a^	0.80 ± 0.15 ^c^	0.91 ± 0.06 ^b^	0.71 ± 0.09 ^d^	ND	ND	ND	ND	ND	ND
C17: 1	0.06 ± 0.01 ^d^	0.55 ± 0.06 ^a^	0.33 ± 0.01 ^b^	0.36 ± 0.01 ^b^	0.28 ± 0.02 ^c^	ND	ND	ND	ND	ND	ND
C18: 0	4.77 ± 0.04 ^h^	11.06 ± 0.25 ^c^	8.68 ± 0.05 ^f^	7.10 ± 0.05 ^g^	12.06 ± 0.03 ^b^	6.39 ± 0.13 ^g^	9.06 ± 0.28 ^ef^	10.35 ± 0.26 ^cd^	13.17 ± 0.22 ^a^	9.75 ± 0.31 ^de^	10.83 ± 1.28 ^c^
C18: 1n-9c	31.09 ± 0.01 ^b^	14.45 ± 0.40 ^i^	17.26 ± 0.70 ^g^	21.58 ± 0.16 ^d^	17.60 ± 0.02 ^g^	33.17 ± 0.37 ^a^	15.32 ± 0.37 ^h^	27.99 ± 0.31 ^c^	20.02 ± 0.45 ^e^	19.11 ± 0.56 ^f^	21.81 ± 0.38 ^d^
C18: 2n-6c	38.19 ± 0.03 ^a^	6.81 ± 0.17 ^g^	13.24 ± 0.14 ^c^	8.48 ± 0.03 ^f^	10.10 ± 0.06 ^e^	29.48 ± 0.23 ^b^	13.20 ± 0.25 ^c^	11.67 ± 0.29 ^d^	4.36 ± 0.10 ^i^	8.77 ± 0.39 ^f^	4.72 ± 0.26 ^h^
C20: 1	6.62 ± 0.01 ^a^	1.38 ± 0.08 ^f^	2.16 ± 0.09 ^e^	1.17 ± 0.05 ^fg^	1.47 ± 0.03 ^f^	1.47 ± 0.07 ^f^	1.02 ± 0.08 ^g^	2.73 ± 0.30 ^d^	3.02 ± 0.17 ^d^	3.44 ± 0.16 ^c^	5.20 ± 0.43 ^b^
C18: 3n-3	ND	ND	ND	ND	ND	0.84 ± 0.01 ^e^	2.61 ± 0.16 ^a^	1.47 ± 0.14 ^d^	0.94 ± 0.15 ^e^	1.91 ± 0.13 ^b^	1.69 ± 0.20 ^c^
C20: 2	0.10 ± 0.01 ^c^	0.60 ± 0.07 ^a^	0.23 ± 0.03 ^b^	0.21 ± 0.00 ^b^	0.21 ± 0.01 ^b^	ND	ND	ND	ND	ND	ND
C22: 0	0.74 ± 0.03 ^b^	0.56 ± 0.07 ^c^	0.24 ± 0.01 ^d^	0.21 ± 0.02 ^d^	0.18 ± 0.01 ^d^	1.21 ± 0.11 ^a^	ND	ND	ND	ND	ND
C22: 1n-9	0.19 ± 0.00 ^f^	1.98 ± 0.08 ^e^	0.56 ± 0.03 ^f^	0.36 ± 0.03 ^f^	0.38 ± 0.03 ^f^	4.06 ± 0.63 ^d^	9.87 ± 0.43 ^b^	6.17 ± 0.38 ^c^	7.05 ± 0.91 ^c^	10.04 ± 0.92 ^b^	11.52 ± 0.98 ^a^
C20: 3n-3	0.15 ± 0.02 ^c^	1.32 ± 0.16 ^a^	1.25 ± 0.09 ^a^	1.11 ± 0.03 ^b^	1.35 ± 0.04 ^a^	ND	ND	ND	ND	ND	ND
C20: 4n-6	ND	ND	ND	ND	ND	0.86 ± 0.44 ^c^	2.78 ± 0.02 ^a^	1.27 ± 0.12 ^c^	2.15 ± 0.21 ^b^	2.30 ± 0.80 ^a^	2.44 ± 0.34 ^ab^
C23: 0	0.09 ± 0.01 ^d^	0.73 ± 0.03 ^a^	0.48 ± 0.03 ^b^	0.36 ± 0.03 ^c^	0.33 ± 0.04 ^c^	ND	ND	ND	ND	ND	ND
C20: 5n-3	1.56 ± 0.03 ^i^	10.24 ± 0.34 ^d^	11.30 ± 0.16 ^b^	11.75 ± 0.02 ^a^	10.73 ± 0.08 ^c^	0.69 ± 0.06 ^j^	6.43 ± 0.09 ^e^	2.00 ± 0.10 ^h^	4.80 ± 0.41 ^f^	3.33 ± 0.15 ^g^	3.40 ± 0.35 ^g^
C22: 6n-3	1.80 ± 0.03 ^f^	16.70 ± 0.20 ^a^	14.31 ± 0.24 ^b^	14.11 ± 0.05 ^b^	12.55 ± 0.03 ^c^	0.89 ± 0.10 ^g^	6.02 ± 0.63 ^d^	3.46 ± 0.14 ^e^	3.00 ± 0.19 ^e^	11.78 ± 0.22 ^c^	5.73 ± 1.34 ^d^
∑SFA	19.32 ± 0.08 ^g^	41.06 ± 0.16 ^b^	35.01 ± 0.45 ^e^	35.78 ± 0.07 ^de^	40.88 ± 0.07 ^b^	27.86 ± 0.33 ^f^	36.85 ± 1.04 ^d^	39.33 ± 0.19 ^c^	45.68 ± 0.82 ^a^	35.70 ± 0.36 ^de^	39.54 ± 1.62 ^c^
∑MUFA	38.89 ± 0.02 ^c^	23.27 ± 0.42 ^h^	24.66 ± 0.68 ^g^	28.56 ± 0.12 ^f^	24.18 ± 0.03 ^g^	39.38 ± 0.33 ^c^	32.12 ± 0.39 ^e^	40.79 ± 0.28 ^b^	39.08 ± 0.71 ^c^	35.59 ± 0.45 ^d^	42.49 ± 0.75 ^a^
∑PUFA	41.69 ± 0.07 ^a^	35.06 ± 0.39 ^c^	40.10 ± 0.43 ^b^	35.45 ± 0.07 ^c^	34.73 ± 0.11 ^c^	32.76 ± 0.30 ^d^	31.04 ± 0.74 ^e^	19.87 ± 0.35 ^g^	15.25 ± 0.13 ^i^	28.71 ± 0.43 ^f^	17.98 ± 1.28 ^h^
n-3	3.50 ± 0.04 ^i^	28.26 ± 0.40 ^a^	26.86 ± 0.33 ^b^	26.97 ± 0.06 ^b^	24.63 ± 0.06 ^c^	2.42 ± 0.16 ^j^	15.06 ± 0.48 ^e^	6.93 ± 0.20 ^h^	8.74 ± 0.37 ^g^	17.01 ± 0.24 ^d^	10.82 ± 1.00 ^f^
n-6	38.19 ± 0.03 ^a^	6.81 ± 0.17 ^hi^	13.24 ± 0.14 ^d^	8.48 ± 0.03 ^g^	10.10 ± 0.06 ^f^	30.34 ± 0.37 ^b^	15.97 ± 0.26 ^c^	12.94 ± 0.38 ^d^	6.51 ± 0.27 ^i^	11.70 ± 0.42 ^e^	7.16 ± 0.41 ^h^
n-6/n-3	10.90 ± 0.12 ^b^	0.24 ± 0.01 ^e^	0.49 ± 0.01 ^e^	0.31 ± 0.00 ^e^	0.41 ± 0.00 ^e^	12.60 ± 0.93 ^a^	1.06 ± 0.02 ^d^	1.87 ± 0.09 ^c^	0.75 ± 0.06 ^de^	0.69 ± 0.03 ^de^	0.66 ± 0.05 ^de^

Note: Data are expressed as mean ± SD. SFA: saturated fatty acids; MUFA: monounsaturated fatty acids; PUFA: polyunsaturated fatty acids. ND: < 0.0033%. Different superscripts letters in the same row indicate significant differences (*p* < 0.05).

**Table 8:** Chromaticity parameters (L*, a*, b*) measured at different anatomical regions of wild and farmed *P. major.*

Location	Indicator	F1	F2	F3	F4	F5	W1	W2	W3	W4	W5	W6
Back Region A	L*	46.18 ± 0.72 ^e^	47.78 ± 1.06 ^e^	45.87 ± 3.11 ^e^	49.25 ± 0.81 ^de^	45.94 ± 2.07 ^e^	52.79 ± 3.51 ^cd^	58.30 ± 2.62 ^a^	53.92 ± 3.84 ^bc^	57.62 ± 2.56 ^ab^	55.15 ± 1.00 ^abc^	53.42 ± 1.11 ^bc^
	a*	−0.8 ± 0.28 ^b^	−1.47 ± 0.26 ^b^	−1.96 ± 0.90 ^b^	−1.24 ± 0.03 ^b^	−1.73 ± 1.07 ^b^	−1.52 ± 1.22 ^b^	1.70 ± 0.55 ^a^	1.87 ± 1.63 ^a^	−0.39 ± 1.15 ^b^	−1.38 ± 0.49 ^b^	−1.47 ± 0.83 ^b^
	b*	1.74 ± 1.73 ^a^	−0.81 ± 0.96 ^b^	−2.63 ± 0.83 ^b^	−2.61 ± 0.64 ^b^	−0.61 ± 0.28 ^b^	−1.60 ± 1.21 ^b^	−2.40 ± 0.67 ^b^	−0.92 ± 0.24 ^b^	−1.46 ± 1.89 ^b^	−4.66 ± 0.92 ^c^	−4.66 ± 0.79 ^c^
Back Region B	L*	47.8 ± 1.08 ^e^	49.91 ± 0.37 ^e^	48.15 ± 0.64 ^e^	50.65 ± 0.57 ^e^	54.33 ± 2.85 ^d^	57.91 ± 2.56 ^bc^	61.95 ± 0.72 ^a^	55.08 ± 2.95 ^cd^	58.44 ± 1.87 ^abc^	60.89 ± 2.39 ^ab^	58.59 ± 3.07 ^abc^
	a*	−0.25 ± 0.64 ^b^	−3.45 ± 2.74 ^c^	−0.98 ± 0.55 ^bc^	0.74 ± 1.17 ^bc^	−1.45 ± 1.84 ^bc^	−1.66 ± 1.78 ^bc^	0.79 ± 0.26 ^ab^	0.79 ± 0.85 ^ab^	1.20 ± 1.67 ^a^	−1.73 ± 1.10 ^bc^	−1.53 ± 1.33 ^bc^
	b*	1.84 ± 2.16 ^a^	0.75 ± 1.64 ^ab^	−2.01 ± 1.36 ^bc^	−0.84 ± 0.67 ^abc^	−0.49 ± 0.20 ^abc^	−2.91 ± 1.78 ^cd^	−1.75 ± 0.57 ^bc^	−1.36 ± 0.61 ^bc^	−0.76 ± 1.90 ^abc^	−6.98 ± 1.86 ^e^	−5.09 ± 1.73 ^de^
Abdomen Region A	L*	73.28 ± 3.01 ^d^	71.39 ± 3.18 ^d^	72.33 ± 2.27 ^d^	75.34 ± 1.53 ^c^	77.91 ± 1.20 ^bc^	80.79 ± 2.19 ^b^	84.68 ± 0.48 ^a^	78.73 ± 2.85 ^bc^	81.38 ± 2.60 ^ab^	78.10 ± 1.77 ^bc^	78.45 ± 0.20 ^bc^
	a*	−2.4 ± 0.13 ^abcd^	−2.09 ± 0.63 ^ab^	−1.55 ± 0.42 ^a^	−2.60 ± 0.61 ^bcd^	−2.76 ± 0.19 ^bcd^	−2.38 ± 0.64 ^abcd^	−3.18 ± 0.16 ^cde^	−2.24 ± 0.87 ^abc^	−2.69 ± 0.32 ^bcd^	−3.93 ± 0.66 ^e^	−3.35 ± 0.72 ^de^
	b*	2.78 ± 1.97 ^bc^	5.55 ± 1.00 ^a^	2.87 ± 1.11 ^bc^	2.33 ± 1.12 ^bc^	0.80 ± 1.90 ^c^	2.07 ± 0.90 ^bc^	0.14 ± 1.22 ^c^	4.61 ± 1.84 ^ab^	0.11 ± 1.49 ^c^	−2.77 ± 1.71 ^d^	−2.59 ± 1.38 ^d^
Abdomen Region B	L*	75.09 ± 1.45 ^d^	77.53 ± 1.57 ^bcd^	72.12 ± 1.39 ^e^	77.38 ± 0.31 ^bcd^	76.59 ± 0.67 ^cd^	78.27 ± 1.04 ^abc^	81.17 ± 0.68 ^a^	80.15 ± 0.92 ^ab^	79.93 ± 2.24 ^ab^	79.47 ± 2.66 ^abc^	79.32 ± 2.08 ^abc^
	a*	−2.18 ± 0.59 ^a^	−3.02 ± 0.26 ^bc^	−1.75 ± 0.66 ^a^	−2.85 ± 0.22 ^bc^	−1.98 ± 0.16 ^a^	−1.94 ± 0.50 ^a^	−2.46 ± 0.06 ^ab^	−1.98 ± 0.20 ^a^	−2.01 ± 0.51 ^a^	−3.18 ± 0.16 ^c^	−3.23 ± 0.13 ^c^
	b*	2.86 ± 1.65 ^ab^	3.75 ± 0.69 ^a^	2.77 ± 1.09 ^ab^	2.26 ± 0.65 ^abc^	1.16 ± 0.95 ^bc^	2.94 ± 1.11 ^ab^	0.48 ± 0.72 ^c^	3.33 ± 1.42 ^a^	1.32 ± 1.22 ^bc^	−2.01 ± 0.83 ^d^	−2.61 ± 0.48 ^d^
Caudal peduncle	L*	61.58 ± 0.90 ^e^	68.49 ± 2.16 ^cd^	61.49 ± 1.68 ^e^	65.36 ± 1.07 ^d^	61.42 ± 2.85 ^e^	65.55 ± 2.82 ^d^	72.44 ± 0.82 ^ab^	71.47 ± 1.23 ^bc^	75.31 ± 2.11 ^a^	73.63 ± 1.30 ^ab^	74.49 ± 2.01 ^ab^
	a*	−0.85 ± 0.93 ^bc^	−2.09 ± 0.48 ^c^	−0.67 ± 0.07 ^bc^	−0.65 ± 0.25 ^bc^	−0.69 ± 0.54 ^bc^	−1.77 ± 1.22 ^c^	0.37 ± 1.13 ^ab^	0.51 ± 1.53 ^a^	−0.80 ± 0.73 ^bc^	−1.39 ± 0.55 ^c^	−1.13 ± 0.44 ^bc^
	b*	5.37 ± 1.53 ^a^	4.02 ± 0.77 ^ab^	2.94 ± 0.73 ^bcd^	2.64 ± 2.12 ^bcd^	1.28 ± 1.62 ^cd^	2.03 ± 1.64 ^bcd^	1.11 ± 0.24 ^cd^	3.52 ± 1.55 ^abc^	1.02 ± 1.10 ^d^	2.53 ± 0.65 ^bcd^	2.68 ± 0.65 ^bcd^
Caudal fin	L*	46.40 ± 2.79 ^a^	32.00 ± 1.37 ^e^	32.91 ± 1.90 ^e^	32.03 ± 0.66 ^e^	32.05 ± 3.38 ^e^	39.22 ± 3.68 ^cd^	40.22 ± 0.88 ^bcd^	35.75 ± 2.74 ^de^	37.63 ± 3.37 ^d^	44.65 ± 2.46 ^ab^	42.38 ± 2.82 ^abc^
	a*	0.04 ± 0.01 ^d^	0.59 ± 0.36 ^d^	1.29 ± 0.27 ^cd^	0.73 ± 0.10 ^cd^	1.20 ± 0.78 ^d^	1.01 ± 1.18 ^d^	2.41 ± 0.16 ^bc^	1.12 ± 0.99 ^d^	3.39 ± 0.80 ^bc^	8.73 ± 0.96 ^a^	7.75 ± 0.64 ^a^
	b*	4.15 ± 1.58 ^cd^	4.03 ± 0.42 ^cd^	3.95 ± 0.57 ^cd^	3.56 ± 0.31 ^d^	6.07 ± 0.44 ^b^	5.74 ± 1.44 ^bc^	3.10 ± 0.07 ^d^	3.45 ± 1.17 ^d^	7.36 ± 1.31 ^b^	9.77 ± 1.10 ^a^	11.28 ± 1.52 ^a^

Note: L*: lightness (0 = black, 100 = white); a*: redness/greenness (positive = red, negative = green); b*: yellowness/blueness (positive = yellow, negative = blue). Data are expressed as mean ± SD. Different superscript letters within the same row indicate significant differences (*p* < 0.05).

## Data Availability

The original contributions presented in the study are included in the article, further inquiries can be directed to the corresponding author.
